# 

**Published:** 1910-01-15

**Authors:** 


### Appointments.

Dr. E. Symington has been reappointed medical officer of health to the Brompton Board of Guardians.

Dr. P. J. O'Connor has been reappointed medical officer of health to the Wigston Urban District Council.

Dr. A. A. Rogers has been appointed medical officer of health to the Itchen Urban District Council.

Dr. J. R. Gillespie, of Belfast, formerly assistant to the medical officer at Cowbridge, Cardiff, has been appointed assistant county medical officer to the Hants County Council.

Dr. Arthur Court has been appointed medical officer and public vaccinator for the Staveley district, under the Chesterfield Board of Guardians. The position was vacant through the resignation of Dr. J. Court, who had filled the office for forty-five years.

Dr. Archibald Kidd, at present a temporary assistant medical officer under the Port of London Authority, has been appointed on the permanent staff as an assistant medical officer of the port.

Dr. Evans, of Cardiff, medical inspector of schools to the Glamorgan Education Committee, has been appointed medical officer to the Pembroke County Council.

Dr. T. L. Drapes has been appointed medical officer of health to the Chepstow Urban District Council.

Dr. James Mair, of Northampton, has been appointed medical officer of health and medical inspector of school children under the Harrogate Town Council.

Dr. D. M. Taylor, deputy medical officer of health and assistant medical officer of schools at York, has been appointed assistant medical officer of schools at Halifax.

Dr. D. Lechmere Anderson has been reappointed medical officer of health to the Doncaster Corporation.

Mr. T. Malcolm Walker has been reappointed medical officer of health under the Banbury Rural District Council.

Dr. Samuel Martyn has been appointed medical officer for the Airdrie Post Office.

Dr. W. A. D. King has been appointed deputy officer at Paignton.

Dr. Andrew John Laird has been appointed medical officer of health to the Cambridge Corporation.

Dr. G. E. Atkins has been appointed medical officer *pro tem.* by the Wombwell Urban District Council.

### Gifts and Bequests.

The following annual subscriptions have been received at the Bank of England for King Edward's Hospital Fund for London :—

| | | | |
|---|---|---|---|
| H.R.H. the Prince of Wales ... ... | £500 | 0 | 0 |
| H.R.H. the Princess of Wales ... | 52 | 10 | 0 |
| H.R.H. Prince Edward of Wales ... | 1 | 1 | 0 |
| H.R.H. Prince Albert of Wales ... | 1 | 1 | 0 |
| H.R.H. Princess Victoria of Wales ... | 1 | 1 | 0 |
| H.R.H. Prince Henry of Wales ... | 1 | 1 | 0 |
| H.R.H. Prince George of Wales ... | 1 | 1 | 0 |
| H.R.H. Prince John of Wales ... | 1 | 1 | 0 |

Mrs. Harriet Paton has left £100 to the Children's Hospital, Paddington Green.

Mr. William John Bone has bequeathed £250 to the Tynemouth Victoria Jubilee Infirmary.

The Middlesex Hospital Cancer Charity has received from Miss Fanny M. Marriage a further donation of £100.

Mr. James Martin, J.P., late of Hove, has left £100 to the Sussex County Hospital and £100 to the Brighton Eye Hospital.

Miss Sarah Dawson has left £500 each to the Stockwell Orphanage, the Medical Missions, Highbury, and £20 to the Victoria Hospital, at Dover.

Mrs. Isabella Graham, of Netherby, has left £500 each to King's College Hospital and to the Hospital for Sick Children, Great Ormond Street, free of legacy duty.

Mr. Edward Henry Cardwell, of Surrey, South Kensington, and Newmarket (racing under the name of Mr. E. Carlton), has left £1,000 to the Royal Surrey County Hospital and £300 to the Cancer Research Association.

The Edinburgh Royal Infirmary has received £1,000, being share of residue of estate bequeathed by the late Mrs. E. J. Patison, of 9 Newbattle Terrace, and a legacy of £100 bequeathed by the late Mr. William Brown, of 22 Cluny Drive.

The treasurer of St. Bartholomew's Hospital has received the following further contributions for the appeal issued in October by the Lord Mayor :—Sir Henry Harben, £1,300; Mr. Henry Perrin, £250; Mr. Henry L. Florence, £105; Mr. R. F. Bayford, £5; Mr. A. J. M. Duncan, £5; Miss Mure, £5.

Sir Hugh Graham, who has taken an active part in organising the emergency typhoid hospital in Montreal, has announced a contribution of £5,000 from Lord Strathcona to the hospital. Lord Strathcona says that he will gladly contribute £20,000 to the fund raised by Montreal citizens for eradicating the causes of these epidemics. Lord Grey has expressed his appreciation of the splendid work accomplished in so short a time.

Mrs. Jane Rosa Haywood, of Kennington Park, S.E., has left £1,000 each to St. Thomas's Hospital, the London Hospital, Whitechapel, the Cancer Hospital, Brompton, the Brompton Hospital for Consumption and Diseases of the Chest, and the Royal Hospital for Incurables, Putney, for the endowment of a bed to be known as the "Jane Rosa Haywood" bed; £1,000 to the British Home for Incurables, Streatham, to endow a bed in memory of "my dear parents" William and Jane Wake; £500 each to the Hospital for Sick Children, Great Ormond Street (for the endowment of a bed to her memory), the London Ophthalmic Hospital; and £250 to the Stockwell Orphanage Convalescent Home, Cliftonville, Margate.

The Surgical Aid Society thankfully acknowledges the following donations :—His Majesty the King (annual subscription), £15 15s.; Her Most Gracious Majesty Queen Alexandra (annual subscription), £10 10s.; G. W. Gandy, Esq., £60; Evelyn Heseltine, Esq., £25; Messrs. Morgan, Grenfell and Co. (annual subscription), £21.

The Tallow Chandlers' Company has given £21 to the London Hospital, £10 10s. each to the East London Hospital for Children, the Middlesex Hospital, the Royal Waterloo Hospital for Children and Women, Guy's Hospital, the Royal Hospital for Incurables, Putney, and St. Bartholomew's Hospital; £5 5s. each to the Great Northern Central Hospital, and the Hospital for Invalid Gentlewomen.

Mr. Henry Hugh Clutton, F.R.C.S., L.S.A., of Portland Place, W., late surgeon to St. Thomas's Hospital, has left estate valued at £46,170 gross and £39,415 net. Mr. Clutton left one daughter, and he directed that if no child of his should attain an interest, or if she should be a daughter who should die without issue, then, subject to her interest, he left such a sum as, with the sums which may have already been given for such purposes by his wife and daughter, shall make up £15,000 to St. Thomas's Hospital Medical School, for furthering the study of the pathological side of clinical medicine and surgery by increasing the stipend of any professor, lecturer, or superintendent who, whilst holding the post, shall not be engaged in private practice. He directed that this fund should be known as the "Clutton Bequest."

### BOOKS RECEIVED.

John Wright and Sons, Ltd., Bristol.

"Open-air at Home." By Stanley H. Bates.
"Lectures on Surgical Nursing." By E. Stanmore Bishop, F.R.C.S.

A. and C. Black.

"The Writers' and Artists' Year-book."
"The Englishwoman's Year-book and Directory."
"Who's Who Year-book," 1910.
"Who's Who," 1910.

### ANSWERS TO CORRESPONDENTS.

T. D. (Glamorgan).—A young fellow, about 30 years of age, subject to fits, thinks if he could get to some home for such cases that he would have a chance to get right. I am writing to ask you if there are any such homes and where I might get a list of the same. His people could manage to pay some little towards his keep in such a home.

[We would suggest you should consult "Medical Homes for Private Patients," published by The Scientific Press, Ltd., 28 and 29 Southampton Street, Strand, London, W.C., price 6d.—Ed. The Hospital.]

### THE QUESTION BOX.

SPECIAL NOTICE.—Questions of interest affecting the honorary medical staff and hospital administration in all departments are invited. When any point arises we hope our readers will formulate it in a question and so co-operate to make this column of real value and interest. In this way the experience of the best-managed hospitals should be made helpful to the whole hospital world. We shall welcome the contribution of both questions and answers.

N.B.—The publication of an answer in this column does not necessarily preclude the publication of subsequent answers to the same question, for a question may involve points which require to be threshed out and fully discussed. Answers, if published, will be paid for at scale rates.

### THE COMMITTEE OF MANAGEMENT AND THE HONORARY MEDICAL STAFF.

### THE METHOD OF REPRESENTATION.

QUESTION: If it is desirable that the Honorary Medical Staff should be members of the Committee of Management, should they all be members *ex officio*, or only some of them? If the latter, how many medical representatives should there be on the lay Committee, and how should they be elected?

### A London Practice.

By Mr. CONRAD W. THIES, Secretary, the Royal Free Hospital.

There is no uniformity in the method of representation adopted at various hospitals, but I will describe that which has been in force at the Royal Free Hospital for some years past, and has proved to be conducive to harmony and the best interests of the institution. The members of the Honorary Medical Staff form the Medical Committee, which meets at such times as may be necessary. The Medical Committee nominate each year two of their members, usually one physician and one surgeon, to serve as their representatives during the ensuing year upon the General Committee of Management (which consists of thirty members), and one of these two members is elected to the Weekly Board (consisting of twelve members). The Medical Committee appoint one of their members as Honorary Secretary, who keeps the minutes of their meetings, and through whom all communications from the Executive are made. All matters which the Medical Committee desire to bring to the notice of the Weekly Board are recorded in a book kept for this purpose. This arrangement ensures that the Medical Committee are promptly informed by their representatives of all subjects under consideration which may directly or indirectly affect their work or interests. It also has the great advantage of interesting the members of the honorary medical staff in the general administrative work of the hospital.

### Manchester Views.

ANSWER: Only limited representation.

By Mr. W. G. CARNT, General Superintendent and Secretary Manchester Royal Infirmary.

I am of opinion that it would be detrimental to the best interests of a hospital for the whole of the members of the Honorary Medical Staff to be members *ex officio* of the Committee of Management. For the reasons I have already given, these gentlemen, to whose skill and ability the institution owes a great deal of its success and reputation, should have a voice in its management, but the administration of a charity built and maintained chiefly by laymen should be chiefly in the hands of laymen. There are many questions to be considered which a lay committee are best capable of considering. The patients are treated by the medical staff, and in case of complaint (and complaints do arise even in the best administered hospitals) it is better for both patients and staff that the Committee of Management (to whom the complaints are made) should be chiefly laymen. They stand between doctor and patient, and public confidence is strengthened in consequence. The subscribers, too, are better satisfied if business men elected by themselves control the finances. For these reasons alone (and there are others) I consider that the Honorary Medical Staff should have only limited representation upon the Committee of Management. A Hospital Committee of from twenty to twenty-four is sufficient for the proper performance of business, and of these not less than two nor more than four should be members of the Honorary Medical Staff. Full physicians and surgeons, and specialists of corresponding rank in the hospital should alone be eligible to serve upon the committee; assistant physicians and assistant surgeons, etc., should not be eligible. These representatives should be elected by the Medical Board, and the rules of the institution should specify that they shall elect at the beginning of each year an agreed number (not less than two nor more than four) to represent them upon the General Committee for a period of twelve months. Those elected should not be eligible for re-election until the whole of the senior staff have in turn served upon the General Committee.

By A HOSPITAL SECRETARY.

The senior members of the Honorary Staff could, upon election, become *ex-officio* members of the General Committee, and the number to be thus elected could be decreed by the rules of the hospital. This system would seem to be the one best calculated to ensure the smooth running and efficiency of a voluntary hospital.

### A Newcastle Practice.

ANSWER: Limited representation.

At the Royal Victoria Infirmary, Newcastle-on-Tyne.

On the House (Management) Committee of the Royal Victoria Infirmary there are four medical and four surgical representatives and one from the out-patient department and one from the special departments, and this form of representation has been in existence for very many years and on the whole works quite satisfactorily.

### A Leicester Practice.

ANSWER: Limited representation.

By Mr. HARRY JOHNSON, House Governor and Secretary, Leicester Infirmary.

All the members of the Hon. Medical Staff are *ex officio* members of the Board of Governors (the business of which is principally confirmatory), but they are not all members of the Weekly Committee, otherwise that body would be unduly large, and the medical representation out of proportion to the lay representation. The rules of this institution therefore provide that the Hon. Medical Staff shall nominate two of their number annually to serve on this Weekly Committee (in addition to the hon. senior physician and hon. senior surgeon, who are *ex officio* members of all committees). The Weekly Committee is the general administrative committee. Similarly with the Finance Committee and the Drug and Medical Stores Committee, the Hon. Medical Staff nominate two of the number for service on these committees (in addition to the hon. senior physician and hon. senior surgeon *ex officio* as above).

### WANTED, A SYSTEM FOR FILING.

QUESTION 1: What is the best system for filing records, documents, and letters suitable for a large hospital?

THE

### GRADUATES' MEDICAL SCHOOLS.

DIARY FOR THE WEEK, JANUARY 17 to 21.

### THE BROMPTON HOSPITAL FOR CONSUMPTION AND DISEASES OF THE CHEST, Fulham Road, S.W.

**At 4 p.m.—Jan. 19,** Dr. Maguire, **Quiescent Lesions of Pulmonary Tuberculosis.**

### ST. JOHN'S HOSPITAL FOR DISEASES OF THE SKIN, Leicester Square, W.C.

**At 6 p.m.—Jan. 20,** Chesterfield Lecture: The Treatment of **Eczema** in all its Forms.

### CENTRAL LONDON THROAT AND EAR HOSPITAL, Gray's Inn Road, W.C.

**At 4.30 p.m.—Jan. 20,** Mr. C. Nourse, **Antral Suppuration.**

### ROYAL SOCIETY OF MEDICINE, 20 Hanover Square, W.

**At 8 p.m.—Jan. 18, Pathological Section:**

*Communications.*—Mr. Walter Edmunds : Action of thyroid and anti-thyroid preparations. Dr. P. J. Cammidge and Dr. Semon : An experimental investigation of the origin and cause of the "pancreatic" (Cammidge) reaction in the urine. Mr. S. G. Shattock : Oöphorectomy and the growth of the pelvis in the cow. Dr. Herbert French : (1) A case of extensive but not complete destruction of the adrenal capsules by caseation without symptoms of Addison's disease. (2) Pigmentary deposits in liver and pancreas in bronzed diabetes. Mr. E. O. Thurston : Chylous cyst from the neck.

**At 5 p.m.—Jan. 20, Dermatological Section:**

*Cases.*—Dr. J. H. Sequeira : Case of tuberculides. Dr. H. G. Adamson : Onychia syphilitica sicca. Dr. E. G. Graham Little : Sclerodermia. And other cases.

**At 8.30 p.m.—Jan. 21, Electro-Therapeutical Section:**

Dr. J. A. Voorthuis (Holland) will demonstrate and read a paper on "The use of the static brush and flame discharge in the treatment of some skin diseases."

### THE HOSPITAL FOR SICK CHILDREN, Great Ormond Street, W.C.

**At 4 p.m.—Jan. 20,** Mr. Corner, **Appendicitis.**

### MEDICAL GRADUATES' COLLEGE AND POLYCLINIC, 22 Chenies Street, W.C.

**At 4 p.m.—Jan. 17,** Dr. Willmott Evans, **Clinique (Skin).**
**At 5.15 p.m.—Jan. 17,** Dr. Essex Wynter, **Heart Disease in Middle Life.**

**At 4 p.m.—Jan. 18,** Dr. Purves Stewart, **Clinique (Medical).**

**At 5.15 p.m.—Jan. 18,** Dr. James Collier, **Acute Encephalitis and Acute Poliomyelitis in the Year 1909.**

**At 4 p.m.—Jan. 19,** Mr. James Cantlie, **Clinique (Surgical).**

**At 5.15 p.m.—Jan. 19,** Dr. Anton Lieven (Aix-la-Chapelle), **On the Treatment of Syphilis.**

**At 4 p.m.—Jan. 20,** Sir Jonathan Hutchinson, **Clinique (Surgical).**

**At 5.15 p.m.—Jan. 20,** Dr. Andrew Wylie, **Foetid Breath.**

**At 4 p.m.—Jan. 21,** Mr. W. Stuart-Low, **Clinique (Ear, Nose. and Throat).**

### NORTH-EAST LONDON POST-GRADUATE COLLEGE, Prince of Wales's General Hospital, Tottenham, N.

**At 4.30 p.m.—Jan. 18,** Dr. E. H. Wallace, **Difficulties in Anæsthesia.**

**At 4.30 p.m.—Jan. 20,** Dr. T. R. Whipham, **Idiocy and Allied Disorders in Children.**

### SOME REFERENCE ANNUALS.

WHO'S WHO, 1910. (London : Adam and Charles Black, Soho Square, London. 10s. 6d. net.)

This nowadays indispensable handbook has been largely amplified, and brought up to date. Its 2,100 odd pages are crammed with information, and many of the short biographies are of interest to medical men.

The same firm issues the following annuals, the 1910 editions of which are excellent in every way :—

"Who's Who Year Book, 1910"—a compendium of useful information, statistical and otherwise; the "Writers' and Artists' Year Book, 1910"—a handbook of reference for the literary free lance; and "The Englishwoman's Year Book," which contains much that is of interest to women workers in all departments. The last contains a helpful article on medical study for women, in which the would-be lady doctor will find many valuable hints, although it must be remarked in passing that the average income of practitioners throughout the country is not £220 a year—would that it were!

